# Influence of pig gut microbiota on *Mycoplasma hyopneumoniae* susceptibility

**DOI:** 10.1186/s13567-019-0701-8

**Published:** 2019-10-28

**Authors:** Meera Surendran Nair, Tyson Eucker, Brian Martinson, Axel Neubauer, Joseph Victoria, Bryon Nicholson, Maria Pieters

**Affiliations:** 1 Department of Veterinary Population Medicine, University of Minnesota, St. Paul, MN USA; 2 Boehringer-Ingelheim Animal Health, Duluth, GA USA

## Abstract

This study investigated the influence of gut microbiome composition in modulating susceptibility to *Mycoplasma hyopneumoniae* in pigs. Thirty-two conventional *M. hyopneumoniae* free piglets were randomly selected from six different litters at 3 weeks of age and were experimentally inoculated with *M. hyopneumoniae* at 8 weeks of age. Lung lesion scores (LS) were recorded 4 weeks post-inoculation (12 weeks of age) from piglet lungs at necropsy. Fecal bacterial community composition of piglets at 3, 8 and 12 weeks of age were targeted by amplifying the V3–V4 region of the 16S rRNA gene. The LS ranged from 0.3 to 43% with an evident clustering of the scores observed in piglets within litters. There were significant differences in species richness and alpha diversity in fecal microbiomes among piglets within litters at different time points (*p* < 0.05). The dissimilarity matrices indicated that at 3 weeks of age, the fecal microbiota of piglets was more dissimilar compared to those from 8 to 12 weeks of age. Specific groups of bacteria in the gut that might predict the decreased severity of *M. hyopneumoniae* associated lesions were identified. The microbial shift at 3 weeks of age was observed to be driven by the increase in abundance of the indicator family, Ruminococcaceae in piglets with low LS (*p* < 0.05). The taxa, *Ruminococcus*_2 having the highest richness scores, correlated significantly between litters showing stronger associations with the lowest LS (r = −0.49, *p* = 0.005). These findings suggest that early life gut microbiota can be a potential determinant for *M. hyopneumoniae* susceptibility in pigs.

## Introduction

*Mycoplasma hyopneumoniae* is the causative agent of enzootic pneumonia and is considered one of the most important bacterial etiologies associated with chronic respiratory illnesses in swine production. Studies on transmission dynamics of *M. hyopneumoniae* indicate the continuous persistence of the bacterium within an infected herd via both sow-to-piglet and horizontal transmission [1]. In endemically infected herds, mature pigs serve as pathogen reservoirs. However, grow-to-finish pigs often develop clinical signs [2] at a high prevalence, which can reach 38–100% in most production systems [3]. *Mycoplasma hyopneumoniae* infections are mostly characterized by non-productive chronic cough lasting weeks to months, and pave an easy path for the establishment of secondary respiratory infections by decreasing the ciliary motility in the respiratory tract [1, 4]. Therefore, *M. hyopneumoniae* associated illness negatively affects profitability and increases production costs, with high morbidity in swine herds.

Controlling mycoplasma-induced pneumonia in the field is a major goal in the swine industry [1, 5]. However, strategies for disease control are not perfect. Farm management, vaccination, medication, and eradication are frequent tools to combat the detrimental effect of *M. hyopneumoniae* infections [6, 7].

Experimental swine models have been historically used to study the pathogenesis, diagnostics, and treatment strategies allied with *M. hyopneumoniae* infection [8, 9]. A plethora of studies over multiple decades have described successful attempts at inducing mycoplasmal pneumonia at experimental level [9, 10]. The associated lung lesions are frequently characterized by purple to grey areas of pulmonary consolidation, mainly observed in the apical and middle lobes, as well as in the accessory and anterior parts of the diaphragmatic lobes [10]. However, variations in pneumonic lesions remain a challenge [9]. Several confounding variables, including possible maternal and paternal genetic effects have been suggested, which could influence the degree of mycoplasmal pneumonia [9, 11, 12]. A recursive partitioning analysis on determinants for experimental enzootic pneumonia reproduction identified study duration, *M. hyopneumoniae* strain, age at inoculation, co-infection with other pathogens, and animal source as the most important factors linked to the observed lung lesion score variability [9]. Conversely, it was observed that even after controlling for many of the aforementioned covariates, the variability in lung lesions exists.

Recent research indicates that changes in gut microbial composition and function can induce alterations in respiratory mucosal immune responses and can lead to disease development in lungs. Several studies in this direction have identified the role of gut microbiota in swine respiratory infections, particularly in porcine reproductive and respiratory syndrome (PRRS), porcine circovirus type 2 (PCV2), and *M. hyopneumoniae* [13–15]. However, mechanisms by which the gut microbiota could affect the pathophysiology of lungs are still in their infancy.

Early-life gut microbiota diversity and composition have been identified as promising predictive biomarkers for health and disease in animals and humans [16–19]. For many years, significant efforts have focused on the function and composition of gut microbiota and their relationship with enteric diseases. To a greater extent, this is due to the close proximity and integral relationship of pathogenic and nonpathogenic microbiota in the digestive tract. Nevertheless, the field of systemic infectious diseases is undergoing a paradigm shift as the significant role of the intestinal microbiome in response to infections outside of the gastrointestinal tract is getting unveiled. For instance, in a pediatric cystic fibrosis study, the patterns of acquisition of “normal microbiome” and the specific assemblages of bacteria in the gut were shown to be associated with exacerbation of respiratory illnesses in infants [18, 20]. The findings contributed to “gut-lung axis” concept, portraying the immunologic cross-talk between the gut and lung mucosa [21]. Therefore, it has been proposed that distinct composition and function of gut flora in early life can influence host susceptibility to respiratory pathogens.

Based on this background information, we conducted an exploratory study to answer whether the gut microbiome composition could modulate susceptibility to *M. hyopneumoniae* infection. The potential association between the presence and abundance of distinct bacterial communities in the piglet gut at different time points, and subsequent susceptibility to develop severe mycoplasma pneumonic lesions was analyzed using the 16S rRNA fecal microbiome profiling of *M. hyopneumoniae* infected pigs.

## Materials and methods

### Ethics statement

Piglets in this study were sampled following protocols approved by Boehringer-Ingelheim Institutional Animal Care and Use Committee and handled according to the nursery farm standard procedures.

### Experimental design and animals

This study consisted of repeated sampling and evaluation of 32 piglets randomly selected from six different litters, over a period of 12 weeks after birth. Piglets were negative for *M. hyopneumoniae* and PRRS virus based on clinical history and lack of seroconversion to both pathogens, as measured by ELISA.

Piglets born to six different dams at a commercial sow farm, irrespective of parity, were weaned at 3 weeks of age, transported to a research facility and were allocated randomly to one of six rooms. All piglets were administered ceftiofur (5 mg/kg, IM) at weaning prior to sample collection, as a part of routine farm practices. All piglets were fed a commercial diet ad libitum. A subset of thirty piglets were housed in five rooms (6 piglets per room) and were intra-tracheally inoculated with *M. hyopneumoniae* strain 232 [22] at a dose of 1 × 10^5^ CCU/mL at 8 weeks of age. Two piglets remained as uninoculated controls throughout the study, and were housed separately in an experimental room. All piglets in this study were monitored for morbidity and mortality, and clinical disease was evaluated and recorded prior to, during, and after *M. hyopneumoniae* inoculation. Fecal swabs were collected from all piglets at 3, 8 and 12 weeks of age, corresponding to weaning, pre-challenge, and 4 weeks post-challenge. All samples were stored at −80 °C until processing.

Piglets were humanely euthanized and necropsied 4 weeks post-challenge, and lungs were removed, and assessed for a total percentage of *M. hyopneumoniae*-suggestive lesions as described in European Pharmacopoeia [10, 23]. Total lung lesion score (LS) was calculated for each pig using the following formula = 100% × [(Right Apical Lobe × 11%) + (Right Cardiac Lobe × 10%) + (Right Diaphragmatic Lobe × 34%) + (Left Apical Lobe × 5%) + (Left Cardiac Lobe× 6%) + (Left Diaphragmatic Lobe × 29%) + (Accessory Lobe × 5%)] [23].

### DNA extraction and 16S amplicon sequencing

Frozen swabs were thawed, vortexed vigorously and total genomic DNA was extracted using DNeasy PowerSoil Kit (Qiagen) according to manufactures’ instructions. The V3–V4 hypervariable region of the microbial 16S rRNA gene was amplified and samples were then processed for MiSeq based sequencing through library generation using the NextEra XT library preparation kit (Cat #FC-131-1096). All samples in the run were barcoded with unique tags on both, the 5′- and 3′-ends, to minimize the chances of bioinformatic misbinning. The library was run on the MiSeq using the 500-cycle kit (Cat # MS-102-2003). High-quality sequences were selected as those containing a median Q-score greater than 20 and trimmed with a cut-off of no more than three uncalled bases at 3′-end or 3-consecutive bases with Q-score measuring less than 16.

### Analysis of microbial community structure and composition in 16S rRNA datasets

DADA2 workflow was used to estimate abundances of bacterial taxa in the entire 16S rRNA dataset collected from different experimental groups [24, 25]. Filtered high-quality sequences obtained from the quality control step were aligned to the SILVA database and trimmed for the alignment region. Chimeric sequences were then removed from the datasets. Filtered sequences were taxonomically classified using the latest release of the SILVA database and contaminating archaeal, mitochondrial, and chloroplast sequences or sequences classified as unknown were removed from further analysis. Subsequently, amplicon sequence variants (ASV) were predicted from these high-quality sequences. Amplicon sequence variants were again mapped to the sequence taxonomy file generated in DADA2 and converted to number of sequences to generate comparative taxonomy data for the datasets. Diversity indices were calculated on the entire 16S rRNA gene sequence data for all samples [26]. The alpha diversity, or the diversity of fecal microbiota within litters, was measured using Shannon index [26]. The compositional similarity between samples from different experimental groups was assessed and compared to the pairwise taxonomic abundances from each litter, against each other, and within the normalized datasets, using Bray–Curtis measure for estimation of beta diversity [27] followed by permutation-based multivariate analysis of variance (PERMANOVA). PERMDISP was used to test the homogeneity of taxonomic dispersion across samples. The distance scores were visualized using principal coordinate analysis (PCoA) plots to reveal the existing compositional segregations among samples. Computation of Bray–Curtis distances and PERMANOVA tests were carried out in vegan package in R (3.6.0 version). Additionally, using a different ordination method called non-metric multidimensional scaling (NMDS), the level of similarity of individual samples within each time point was visualized. Ordiellipses were used to create a confidence ellipse around each of the litter communities and each dashed ellipse represented the 95% confidence interval for the centroid of each stratification group. An algorithm for discriminating high-dimensional biomarker of genomic features, linear discriminant analysis effect size (LEfSe) was used to determine the differences in fecal microbiome at genus level among different litters [28]. Random Forest analysis was applied to identify genera that are important for distinguishing litters [29]. Mean decrease in accuracy (MDA) scores were determined to represent the predictive accuracy that a given feature or genus possess for assigning a sample to a litter representing their LS.

### Statistical analysis

The effect of litters on the alpha diversity and species richness was analyzed using one-way ANOVA, and Tukey’s test was used to perform post hoc comparisons. Further, the effect of litters and time on the bacterial community composition was analyzed using PERMANOVA (adonis function, 99 permutations). The differentially abundant taxa driving the microbial shift between litters were determined by characterizing species indicator values or Indval. Tukey’s test was used to identify changes in the relative abundance of these indicator taxa between litters and the significance was detected at *p* < 0.05. Pearson’s correlation coefficients [30] were used to determine the correlation of relative abundance of amplicon sequence variants with *M. hyopneumoniae* induced lung lesions.

## Results

### Lung lesions

**Figure 1:**
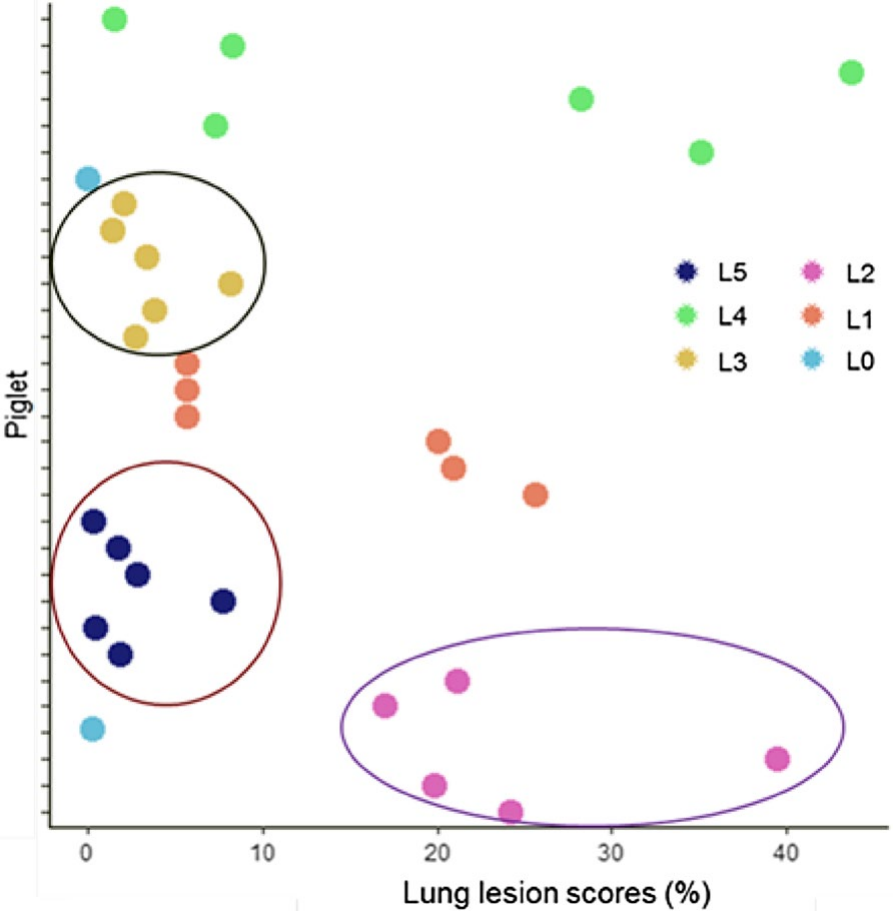
**Lung lesion scores (LS) were plotted against piglet ID.** Lungs were removed 4 weeks post-infection at necropsy and *M. hyopneumoniae*-induced pneumonic lesions were scored as described in European Pharmacopoeia and plotted against piglet ID. Piglets from different litters showed varied susceptibility to *M. hyopneumonaie* challenge. Litters were represented in sequential numbers 0–5. L0 included uninoculated control piglets. Litters 1–5 included all *M. hyopneumonaie* experimentally inoculated piglets. Piglets from L3 and L5 showed the least LS, whereas those from L2 and L4 showed the highest LS.

Lung lesions appeared as confluent, collapsed with purple consolidation, and clearly demarcated from adjacent normal tissue by a sharp line. Lung lesion scores were plotted against piglet IDs and a close clustering of lesion scores within littermates was observed (Figure 1). The plot indicated that although all piglets were challenged with the same strain of *M. hyopneumoniae*, using the same dose intra-tracheally, piglets from different litters showed varied susceptibility to the pathogen. Piglets from two litters showed the highest mean LS of ~20% (95% CI 30.32–10.4) whereas litters from other two litters showed the lowest mean LS of 2.5% (95% CI 4.7–0.31). Henceforth, subsequent microbial community analysis for determining the influence of piglet gut flora composition on the susceptibility to *M. hyopneumoniae* was performed in challenged piglets, using litters as the stratification or grouping variable. Data from uninoculated piglets were included in the analysis as baseline control for growth dependent changes.

### Sequencing of fecal samples

Fecal bacterial community compositions of piglets at 3, 8 and 12 weeks of age were targeted by amplifying the V3–V4 region of 16S rRNA gene. After quality filtering and demultiplexing, 1 415 958 clean reads were obtained with an average of 15 997 assigned to 389 different ASVs (Additional file 1). Rarefaction curves of these indices plateaued a saturation phase after 1000 reads per sample with Good’s coverage of >90% for all samples, suggesting adequate reads and sequencing depth for the microbiota investigation (Additional file 2). Low abundance ASVs under 3% cutoffs were removed prior to compositional and LEfSe analyses. The reads of samples were also normalized using cumulative sum scaling for downstream analysis.

### Diversity analysis on fecal microbiome composition and abundance

**Figure 2:**
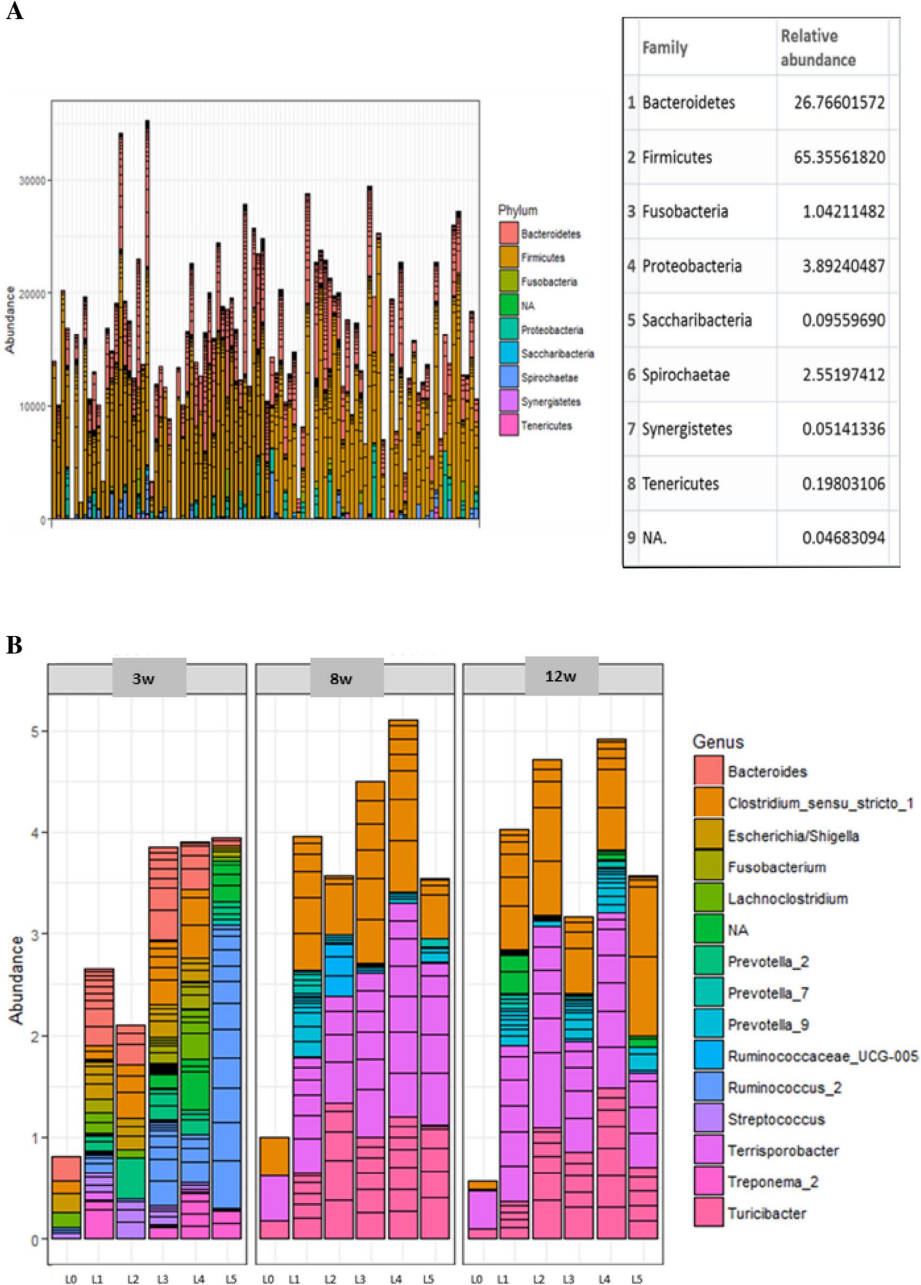
**Taxonomic distribution of fecal microbiome of piglets. A** The majority of bacterial species identified in all fecal samples belonged to the phylum Firmicutes, followed by Bacteroidetes and Proteobacteria, irrespective of the litters. **B** Top 20 genera present in different litters over the course of experiment (3 w, 8 w, 12 w = 3, 8 and 12 weeks of age, respectively). Litters were represented in sequential numbers 0–5. L0 included uninoculated control piglets. Litters 1–5 included all *M. hyopneumonaie* experimentally inoculated piglets.

**Figure 3:**
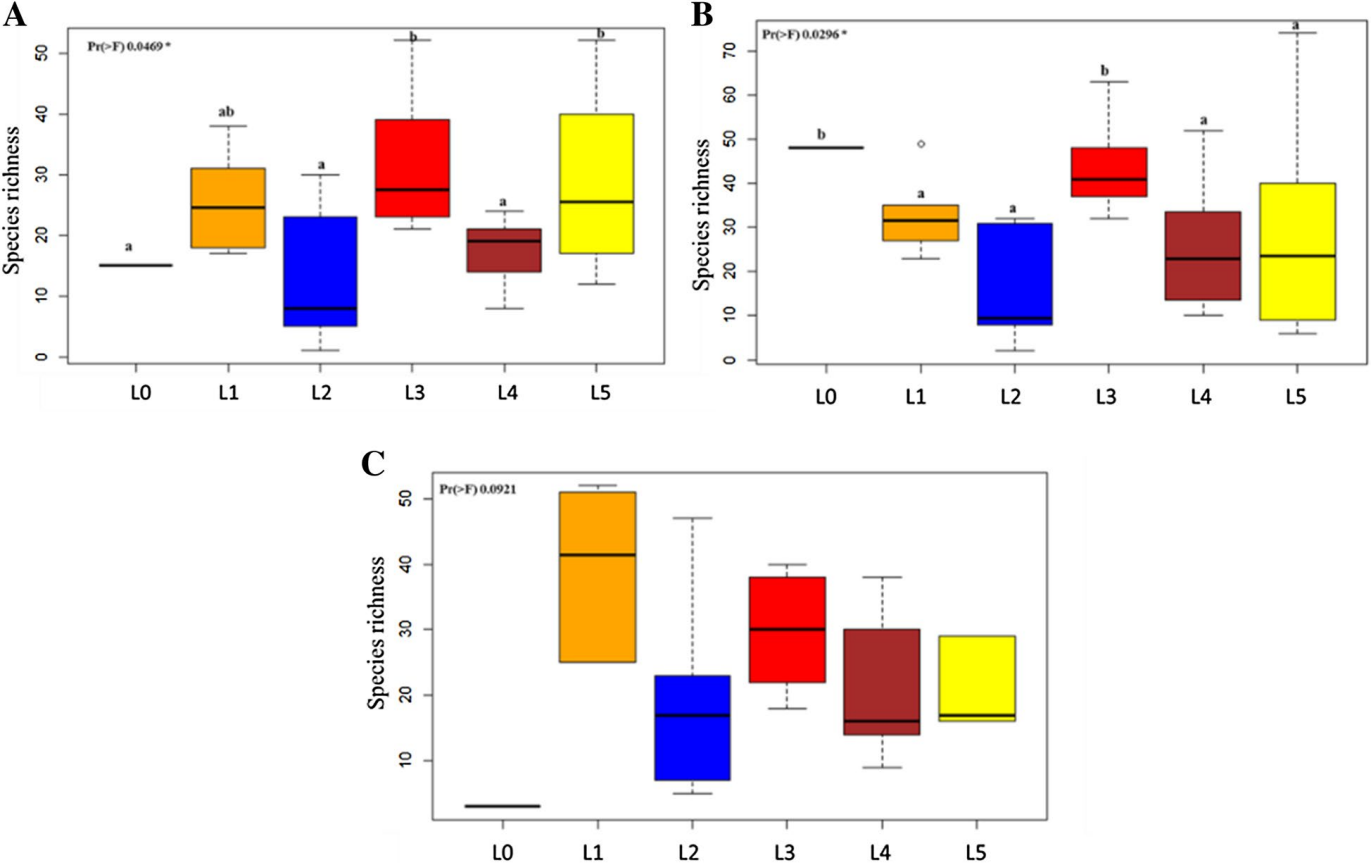
**Variations in species richness among pig fecal samples.** Comparison of the number of observed species (species richness) among piglets from different litters were performed using one-way ANOVA and Tukey’s test was used to perform post hoc comparisons. Litters were represented in sequential numbers 0–5. L0 included uninoculated control piglets. Litters 1–5 included all *M. hyopneumonaie* experimentally inoculated piglets (Black: L0, orange: L1, blue: L2, red: L3, maroon: L4, yellow: L5). Piglets from L3 and L5 showed the least LS, whereas those from L2 and L4 showed the highest LS. Figures depict differences in species richness at weeks 3 (**A**), 8 (**B**), and 12 (**C**). The average distribution of number of species was measured using the entire amplicon sequence variant dataset. The lines inside boxes represent the mean and superscripts with different letters are significantly different from each other (*p* <  0.05).

Firmicutes, Bacteroidetes, Proteobacteria, Fusobacteria, and Spirochaetes were the most abundant phyla and accounted for over 99% of all piglet microbial communities (Figure 2A). LEfSe algorithm on microbial abundances at genus level in piglets showed significant variations between different litters. Figure 2B depicts the top 20 genera present in different litters over the course of the experiment. Among challenged piglets, the observed number of species or species richness was significantly higher in litters with low LS compared to litters with high LS at 3 weeks of age (*p* < 0.05; Figure 3). Additionally, a significant difference in species richness between one of the litters with high LS and all others persisted at 8 weeks of age (*p* < 0.05; Figure 3).

**Figure 4:**
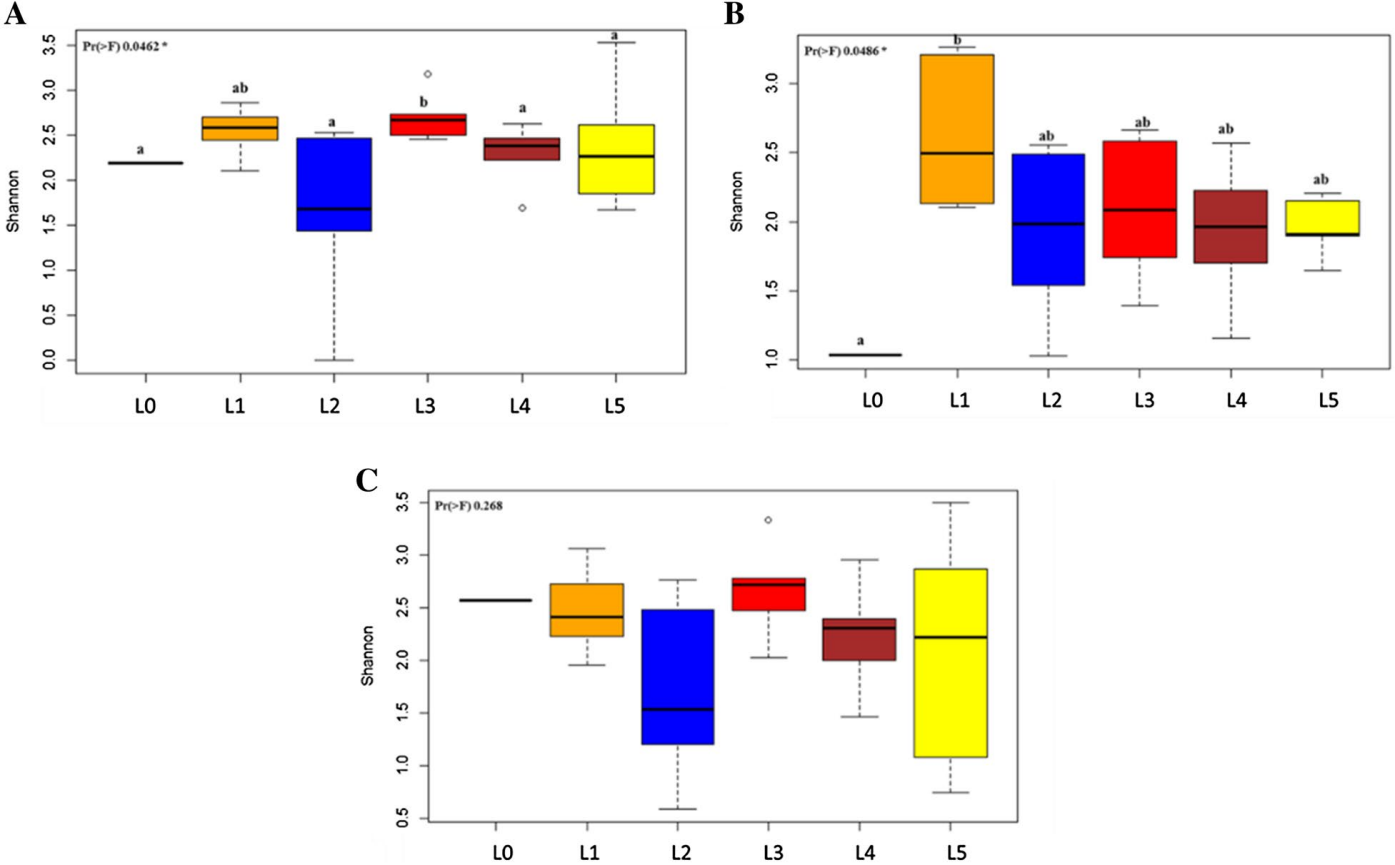
**Alpha diversity of pig fecal samples.** Comparison of Shannon diversity index scores were shown for weeks 3, 8 and 12 in panels **A**, **B** and **C** respectively. The alpha diversity metrics were calculated using the entire amplicon sequence variant dataset. The lines inside boxes represent the mean and superscripts with different letters are significantly different from each other (*p* <  0.05). Comparison of Shannon diversity indices among piglets from different litters (Black: L0, orange: L1, blue: L2, red: L3, maroon: L4, yellow: L5) were performed using one-way ANOVA and Tukey’s test was used to perform post hoc comparisons. Litters were represented in sequential numbers 0–5. L0 included uninoculated control piglets. Litters 1–5 included all *M. hyopneumoniae* experimentally inoculated piglets. Piglets from L3 and L5 showed the least LS, whereas those from L2 and L4 showed the highest LS.

The Shannon index measure accounted for the level of distribution of the observed species in the fecal samples. There was a significant difference in microbial diversities between litters, which was more evident in piglets at 3 weeks of age (*p* < 0.05; Figure 4). The microbial diversity in litters with high LS was significantly lower compared to that of a litter with low LS at early age (*p* < 0.05; Figure 4A). Nevertheless, as piglets moved to the nursery, and finally to the finisher phase, the differences in microbial diversities were less apparent (Figures 4B and C). Thus, it was observed that a highly diverse microbial community during early life was an evident characteristic of piglet fecal microbiota. The litter with the lowest microbial diversity showed the highest severity with *M. hyopneumoniae* lesions later in life.

### Shifts in gut microbial diversity and abundance in piglets from different litters

**Figure 5:**
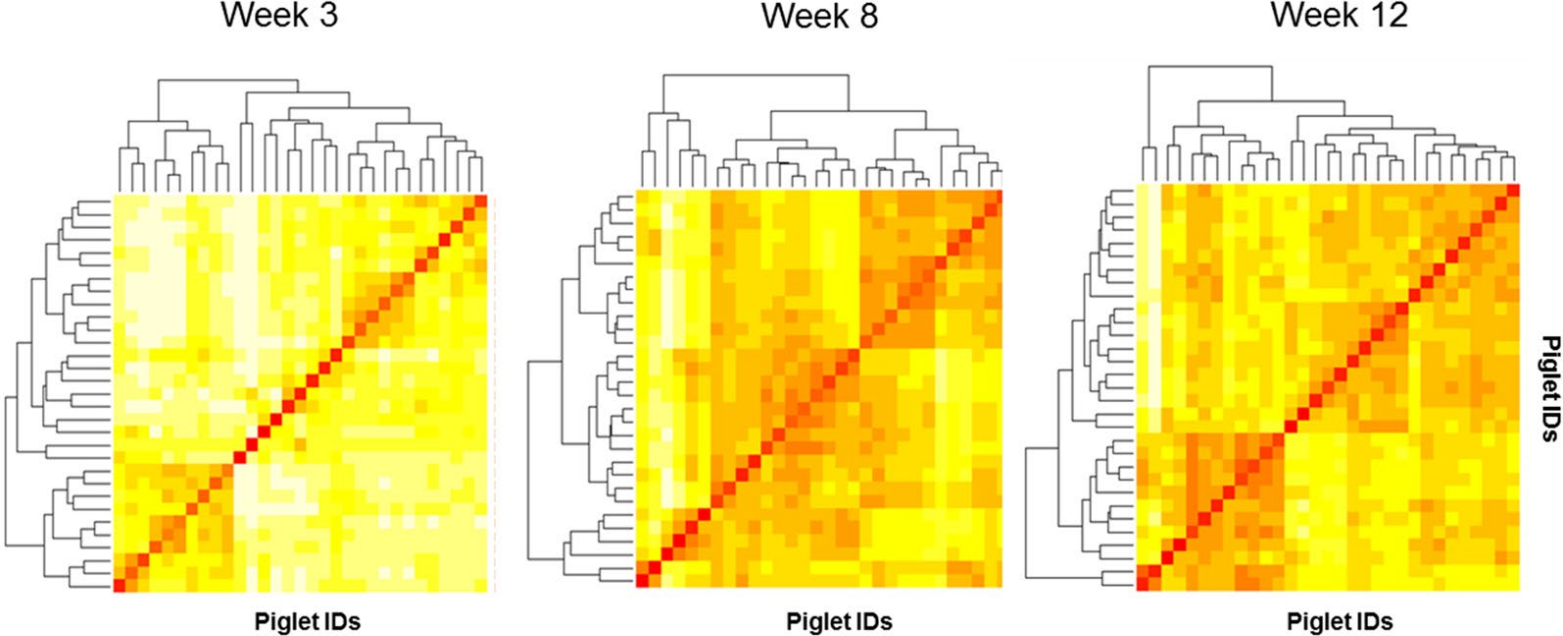
**Microbial differential diversity among piglet fecal samples.** In the heat map, each small square represents one sample. The more intense the color in the heat map, the more similar the pair of pig fecal samples. Results indicate that at week 3, the piglet fecal samples from different litters were more dissimilar compared to those from weeks 8 to 12 (more intense red color).

Using the Bray–Curtis distance matrix, the differential diversity (beta diversity) between samples [26], both in terms of presence/absence and abundance of taxa was identified (Figure 5). The more intense the color in the heat map, the more similar a pair of samples was. The dissimilarity matrices indicated that at 3 weeks of age, piglet fecal samples were more dissimilar compared to those from 8 to 12 weeks of age. Additionally, PCoA analysis revealed significant segregations among samples, indicating a time-dependent separation in fecal microbial community composition, and abundance (Additional file 3). The temporal effects on the clustering contributed to 22% of variability among samples.

**Figure 6:**
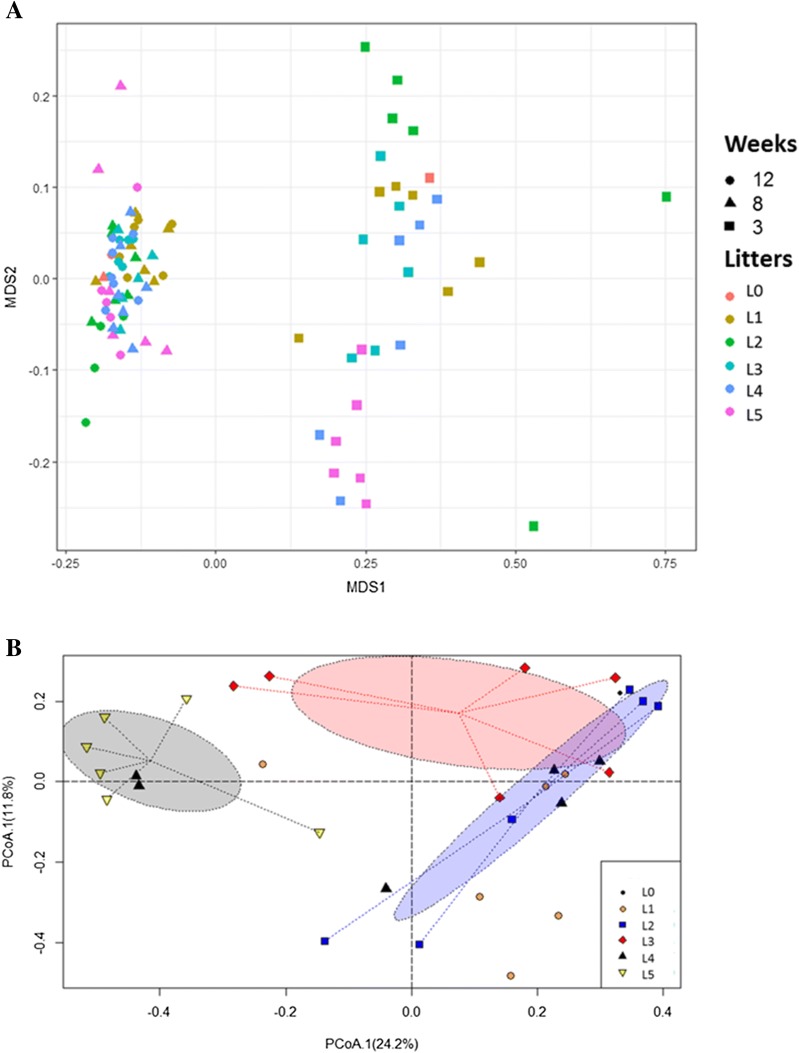
**Beta diversity indices. A** Non-metric multidimensional scaling of Bray–Curtis distances between fecal samples based on microbial abundances. The stress associated with this ordination was 0.091. Points are colored by litters to which the samples belonged, and shape of each point indicates the age of pigs in weeks. L0 included uninoculated control piglets. Litters 1–5 included all *M. hyopneumoniae* experimentally inoculated piglets. **B** Each dashed ellipse represents the 95% confidence interval for the centroid of each litter as calculated by ordiellipse. At week 3, centroids were significantly separated among L2 (blue), L3 (red) and L5 (gray) litters. Piglets from L3 and L5 showed the least LS, whereas those from L2 and L4 showed the highest LS.

As shown in the NMDS plot depicted in Figure 6A, samples at 3 weeks of age diverged from each other whereas those from 8 to 12 weeks of age clustered in a similar pattern. Subsequently, clustering patterns on week 3 were analyzed using ordiellipses. Although there were overlaps in different litters (Additional file 4) at 3 weeks of age, centroids of litters with low LS were significantly separated from other litters, indicating a core division in the fecal bacterial communities of piglets (Figure 6B). Piglets showing high LS after *M. hyopneumoniae* infection showed a different microbial composition prior to weaning compared to those piglets showing decreased LS.

**Figure 7:**
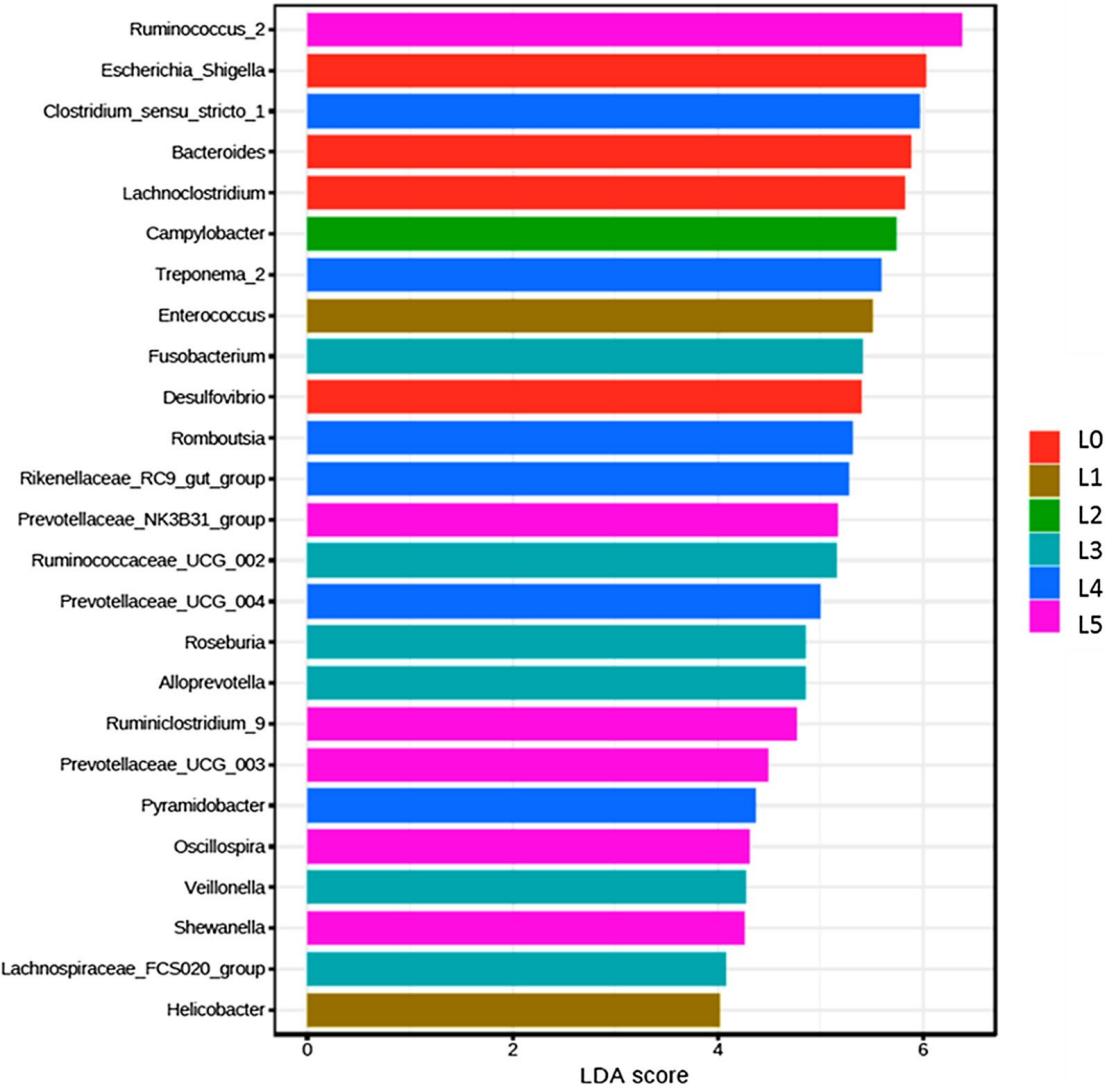
**Differences in phylotypes of early life gut microbiota corresponding to different litters were identified using LEfSe.** Histogram of the LDA scores computed for features differentially abundant (significant threshold > 2 fold and *p*  <  0.05) among litters was shown. The enriched taxa in different litters are shown in different colors. L0 included uninoculated control piglets. Litters 1–5 included all *M. hyopneumoniae* experimentally inoculated piglets. Piglets from L3 and L5 showed the least LS, whereas those from L2 and L4 showed the highest LS.

Twenty-five phylotypes were identified at genus level as high-dimensional biomarkers in different litters at the age of 3 weeks (Figure 7). Twenty-five percent of these phylotypes were relatively enriched in one of the litters with the lowest LS and the majority of these significantly abundant taxa belonged to short-chain fatty acid (SCFA) producing bacterial genera including, *Ruminococcus*_2, Prevotellaceae_NK3B31_group, *Ruminiclostridium*_9, Prevotellaceae_UCG_003, and *Oscillospira*. Similarly, in the second litter with low LS, higher abundance of Ruminococcaceae_UCG_002, *Roseburia*, *Fusobacterium*, *Alloprevotella*, Lachnospiraceae_FCS020_group, and *Veillonella* was observed.

**Figure 8:**
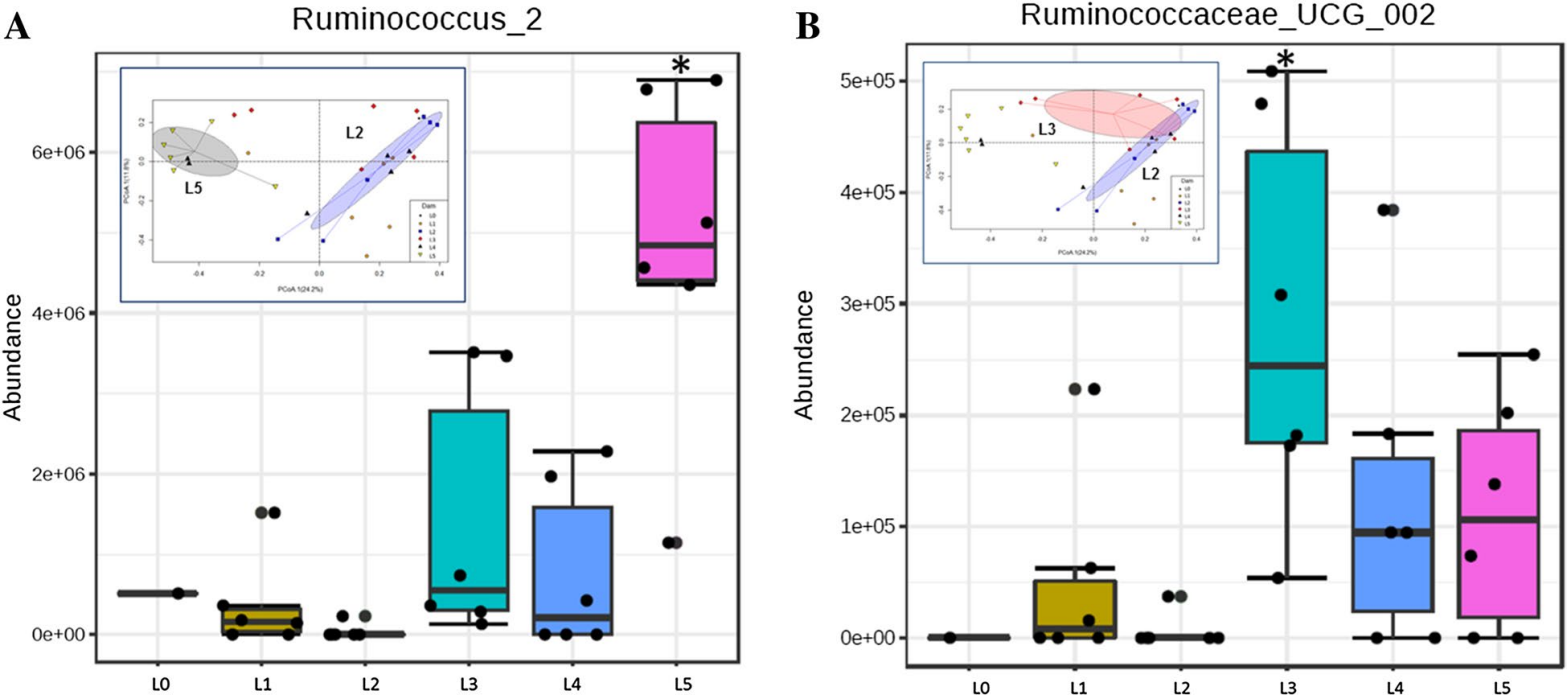
**Microbial shifts between litters and taxa driving the shifts. A** The community shifts between L2 and L5 at week 3 was mainly driven by increase in abundance of the indicator species, *Ruminococcus*_2. L0 included uninoculated control piglets. Litters 1–5 included all *M. hyopneumoniae* experimentally inoculated piglets. **B** Similarly, *Ruminococcaceae*_UCG_002 drove the shift between L2 and L3. The inserted ordiellipse plots indicate significant separation between the L2 (high LS) and L5 (low LS) in **A**, and separation between L2 and L3 (low LS) in **B**.

Using Random Forest analysis, it was identified that the taxon *Ruminococcus*_2 was the most discriminative genus in one of the litters with low LS whereas that in second litter with low LS was Ruminococcaceae_UCG_002. As shown in Additional file 5, the more important a genus is to classifying samples into specific litter, the further to the right its point is on the graph and highest its predictive value. Finally, the differentially abundant taxa driving the microbial shift between litters were determined. In line with the Random Forest prediction algorithm, species indicator analysis revealed that the observed microbial shift at 3 weeks of age was mainly driven by the increase in abundance of the indicator family, Ruminococcaceae in litters with low LS (*p* < 0.05; Figure 8).

### Genera associated with lung lesion scores

**Figure 9:**
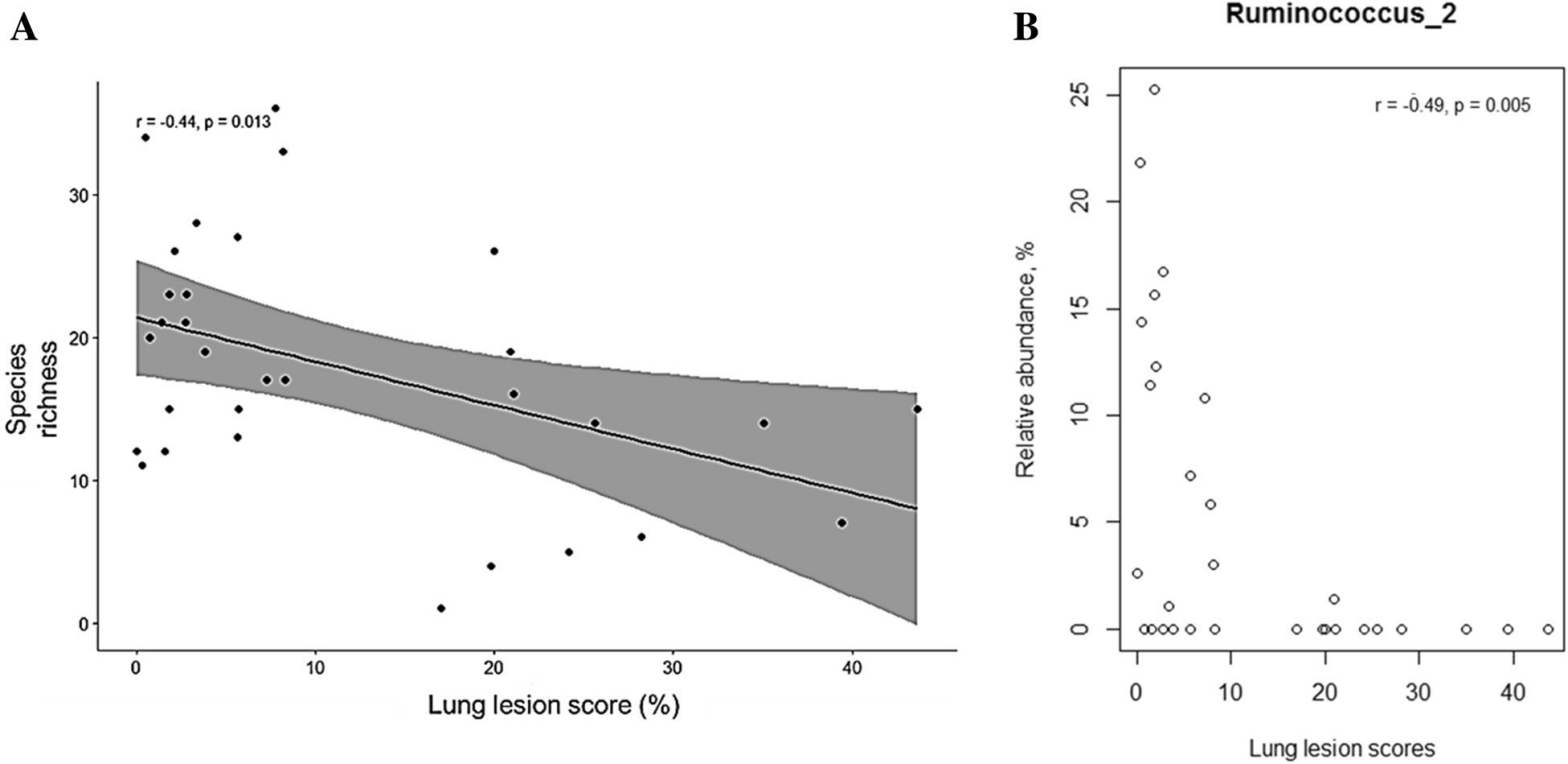
**Correlation between fecal microorganisms and lung lesion scores (LS). A** A significant negative correlation was observed between richness counts determined at week 3 and LS (r = −0.44, *p* = 0.013). **B** The week 3 relative abundance of genus, *Ruminococcus*_2 was negatively correlated with LS (r = −0.49, *p* = 0.005).

A significant negative correlation was observed between richness counts determined at 3 weeks of age and LS recorded (r = −0.44, *p* = 0.013; Figure 9A). The relative abundance of the genus, *Ruminococcus*_2 was strongly correlated with piglets showing lowest LS (r = −0.49, *p* = 0.005; Figure 9B). There were no apparent correlations between richness counts at 8 and 12 weeks of age and LS.

## Discussion

This study delineated a potential association between the presence and abundance of distinct bacterial communities in the piglet gut at 3 weeks of age, and subsequent susceptibility to develop severe mycoplasma pneumonic lesions. The greatest inter-individual variability in the gut microbiota occurred in the first 3 weeks of life, after that, piglets from different litters were similar to others in terms of fecal microbial patterns. In addition, specific groups of bacteria in the gut that might predict the decreased severity of *M. hyopneumoniae* associated respiratory complications were identified. At 3 weeks of age, the majority of significantly abundant taxa in piglets who developed low LS belonged to SCFA producing taxon, Ruminococcaceae. However, the variability of the microbial patterns in early life and its relationship to *M. hyopneumoniae* exposures highlights prospective opportunities for both nutritional and therapeutic interventions to control respiratory disease in pigs.

Various recent studies have investigated the immediate and long-lasting effects of early-life antibiotic administration on pig intestinal microbiota and gut functionality [16, 31, 32]. Nevertheless, studies are lacking in similar direction determining the effect of gut dysbiosis on respiratory infections in pigs. Therefore, better understanding on the fallouts of disturbances in gut microbiota could elucidate the susceptibility to swine respiratory diseases.

Studies on human airway diseases are now replete with correlations between the gut microbiome and chronic airway ailments [16, 17]. However, many of these findings are correlative and associative, which does not necessarily imply a causal effect. Therefore, to set the path from correlation to causation in swine respiratory diseases, knowledge on molecular mechanisms coupled with longitudinal microbiota-based studies are needed.

This observational study lays the groundwork for larger studies to better characterize the intestinal microbiome in pigs with enzootic pneumonia and the interrelationship between the gut and the respiratory tract in disease progression. Consistent with previous findings, the majority of bacterial species identified in this study belonged to the phylum Firmicutes, followed by Bacteroidetes and Proteobacteria, irrespective of the litters throughout the course of the study [33–35]. It was an interesting observation that even after controlling for bacterial strain, dose, and route of infection, the severity of infection was explained by the litters to which the piglets were born. Litters with low microbial diversity in the gut during early life (3 weeks of age) showed severe LS on exposure to *M. hyopneumoniae*. Nevertheless, infection did not seem to have a profound influence on the composition of the gut. Our findings thus suggest that the gut microbiome in early life could be a determinant of respiratory disease progression in pigs.

Additionally, there could be maternal or host genetic effects impacting the fecal microbiota composition. Recent gut microbiome studies in human subjects, as well as in pigs, unraveled evidences on host genetic profiles and their links to differences in microbiome composition, suggesting the role of host genetics shaping the gut microbiome [36, 37].

A strong negative correlation had also been identified between low species richness in the gut at the age of 3 weeks and increased susceptibility to *M. hyopneumoniae* later in the course of the study. However, as early as 8 weeks after birth, these differences in diversity were lost. Prophylactic parenteral administration of antibiotics pre-weaning, shared feeding and housing conditions could be attributed to the change observed in the post-weaning piglet gut microbial populations. This proposes the presence of an interventional window early in life with dietary, antibiotic, probiotic or oral modulatory regimens. Similar to what was observed in this study, there are reports which suggest that respiratory diseases such as asthma, chronic obstructive pulmonary disease and cystic fibrosis in humans have been associated with decreased microbial diversity [38, 39], linking the aberrations in the gut microbiota to infectious and immune-mediated diseases [40]. Likewise, previous studies have shown that early life gastrointestinal microbial diversity is critical for an appropriate systemic immune function later in life [13, 41–44].

Specific groups of bacteria in the gut that might predict the decreased severity of *M. hyopneumoniae* associated respiratory complications were identified, implicating, perhaps, a role for specific microbial taxa in lung health and disease progression in pigs. LEfSe algorithm on microbial abundances at genus levels in piglets showed significant differences between different litters. For instance, at 3 weeks of age, the majority of significantly abundant taxa in piglets from a litter that developed low LS belonged to SCFA producing taxa. This observation was noteworthy as recent literature has evidenced on the key role for SCFAs in the regulation of human respiratory diseases [45, 46]. Short-chain fatty acids including acetate, butyrate, and propionate, are the end-products of dietary fiber fermentation by commensal gut flora and have been suggested to be the vital nexus between microbiota and different host organ systems [47]. Studies have reported that in addition to having local effects in the gut, SCFAs enter the circulation, modulate bone-marrow hematopoiesis and thereby could promote regulatory or pro-inflammatory responses in the lung [41, 48, 49]. Thus, SCFAs can contribute to the homeostatic immunological environment in the respiratory tract and influence the severity of inflammation in the lung.

In the fecal samples, a significant increase in the abundance of *Ruminococcus* prior to *M. hyopneumoniae* inoculation was observed and they were indeed the indicator species that drove the microbial shift between litters with high and low LS. Other SCFA producing genera identified differentially expressed in litters with low LS were *Prevotella*, *Ruminiclostridium,* and *Oscillospira*. Among them, *Ruminiclostridium* is an acetate producer [50] whereas *Prevotella* and *Oscillospira* are butyrate producers [51].

The genus *Ruminococcus* belongs to the Clostridiales order and is known to produce the SCFAs acetate, and butyrate. The increased abundance of members of Ruminococcaceae in the gut and decreased incidence of interstitial pneumonia was previously reported in a study evaluating the role of the microbiome in PRRSV and PCV-2 viruses co-infection in pigs [50]. The presence of *Ruminococcus* in the gut has also been viewed as positive markers for weight gain in farm animals [51]. Another report assessed the role of early gut microbiota composition of piglets in modulating the susceptibility to post-weaning diarrhea [19]. Compared to diarrheic piglets, a higher abundance of Ruminococcaceae, Prevotellaceae, Lachnospiraceae, and Lactobacillaceae was observed in the gut microbiota of healthy piglets [19]. Thus, the knowledge on host–microbial relationships, accrued through this study could be used for future research to define and characterize the composition and function of a “healthy” pig gut microbiota to successfully implement *M. hyopneumoniae* control strategies. In this era of antimicrobial resistance, world health organizations are in fact urging national institutions to develop strategies to replace the usage of antimicrobial drugs in animal agriculture with immunomodulatory strategies. Therefore, this type of investigations will prove invaluable as we work to modulate the microbiota of agricultural animals for future sustainable animal production.

Follow up studies are currently underway including information on dam microbiota and cross-fostering as covariates to explain the observed clustering among littermates and variation between different litters. Moreover, mechanistic studies to better understand the existence of immunologic and metabolic cross-talk between SCFA producing microbiota in the gut and *M. hyopneumoniae* colonization in the lungs are highly warranted. In order to overcome one other limitation of the current study that only piglets from a single genetic line were included, prospective studies are also needed to be designed to address the piglet genetic background variations on gut microbial compositions and susceptibility to *M. hyopneumoniae.*

Briefly, in this study, it was identified that the early life piglet microbiome was strongly correlated to the variability in *M. hyopneumoniae* induced LS. Thus, it is hypothesized that a characteristic assemblage of gut microbiota could bridge the function of the two organ systems in this population.

## Supplementary information

**Additional file 1. Reads summary of pig fecal samples.**

**Additional file 2. Rarefaction curves of the sequencing data**. The observed species of each sample was in plateaued saturation phase over 1000 reads. Each color indicates a litter and sample size denotes the reads length/per sample. Litters were represented in sequential numbers 0–5. L0 included uninoculated control piglets. Litters 1–5 included all *M. hyopneumonaie* experimentally inoculated piglets.

**Additional file 3. Difference in bacterial composition among different samples.** The PERMANOVA results showed that the microbiome composition in samples was influenced by age (*p* = 0.01). Litters were represented in sequential numbers 0–5. L0 included uninoculated control piglets. Litters 1–5 included all *M. hyopneumonaie* experimentally inoculated piglets.

**Additional file 4. Samples cluster by litters at week 3 in PCoA plots.** Each dashed ellipse represents the 95% confidence interval for the centroid of each stratification group. Litters were represented in sequential numbers 0–5. L0 included uninoculated control piglets. Litters 1–5 included all *M. hyopneumonaie* experimentally inoculated piglets. Piglets from L3 and L5 showed the least LS whereas those from L2 and L4 showed the highest LS.

**Additional file 5. Differences in phylotypes of early life gut microbiota corresponding to different litters were identified Random Forest analysis**. Using the Random Forest classifier, the most discriminative genus-level taxa between litters were identified. The taxa are ranked by the mean decrease in classification accuracy when they are permuted. The mean decrease accuracy is a measure of predictive power. The value indicates how much predictive power is lost if a given genus is removed or permuted in the Random Forest algorithm while classifying samples into litter groups. Litters were represented in sequential numbers 0–5. L0 included uninoculated control piglets. Litters 1–5 included all *M. hyopneumonaie* experimentally inoculated piglets.

## Data Availability

Data are proprietary to Boehringer Ingelheim Animal Health (BIAH). Reasonable requests for access to the raw data will be considered by the authors with permission from BIAH.
