# Supplementary material for: Influence of pig gut microbiota on Mycoplasma hyopneumoniae susceptibility
Source: Vet Res. 2019 Oct 28;50:86. doi: 10.1186/s13567-019-0701-8 (PMC6819593; doi:10.1186/s13567-019-0701-8)
Supplement: Supplementary file 1 — Additional file 1. Reads summary of pig fecal samples. [file 13567_2019_701_MOESM1_ESM.docx]

| **Litter Groups** | **Total reads** | **Average number of reads assigned to ASVs** | **Number of assigned ASVs** |
| --- | --- | --- | --- |
| L0 | 55 799 | 18 600 ± 11 456 | 75 |
| L1 | 342 855 | 19 047 ± 4936 | 391 |
| L2 | 196 736 | 10 929 ± 6587 | 209 |
| L3 | 290 084 | 16 115 ± 7031 | 394 |
| L4 | 300 649 | 14 316 ± 7551 | 365 |
| L5 | 285 634 | 15 868 ± 8453 | 389 |
| Total | 1 415 958 | 15, 997 | 398 |
