# Supplementary figures and images for: Influence of pig gut microbiota on Mycoplasma hyopneumoniae susceptibility
Source: Vet Res. 2019 Oct 28;50:86. doi: 10.1186/s13567-019-0701-8 (PMC6819593; doi:10.1186/s13567-019-0701-8)

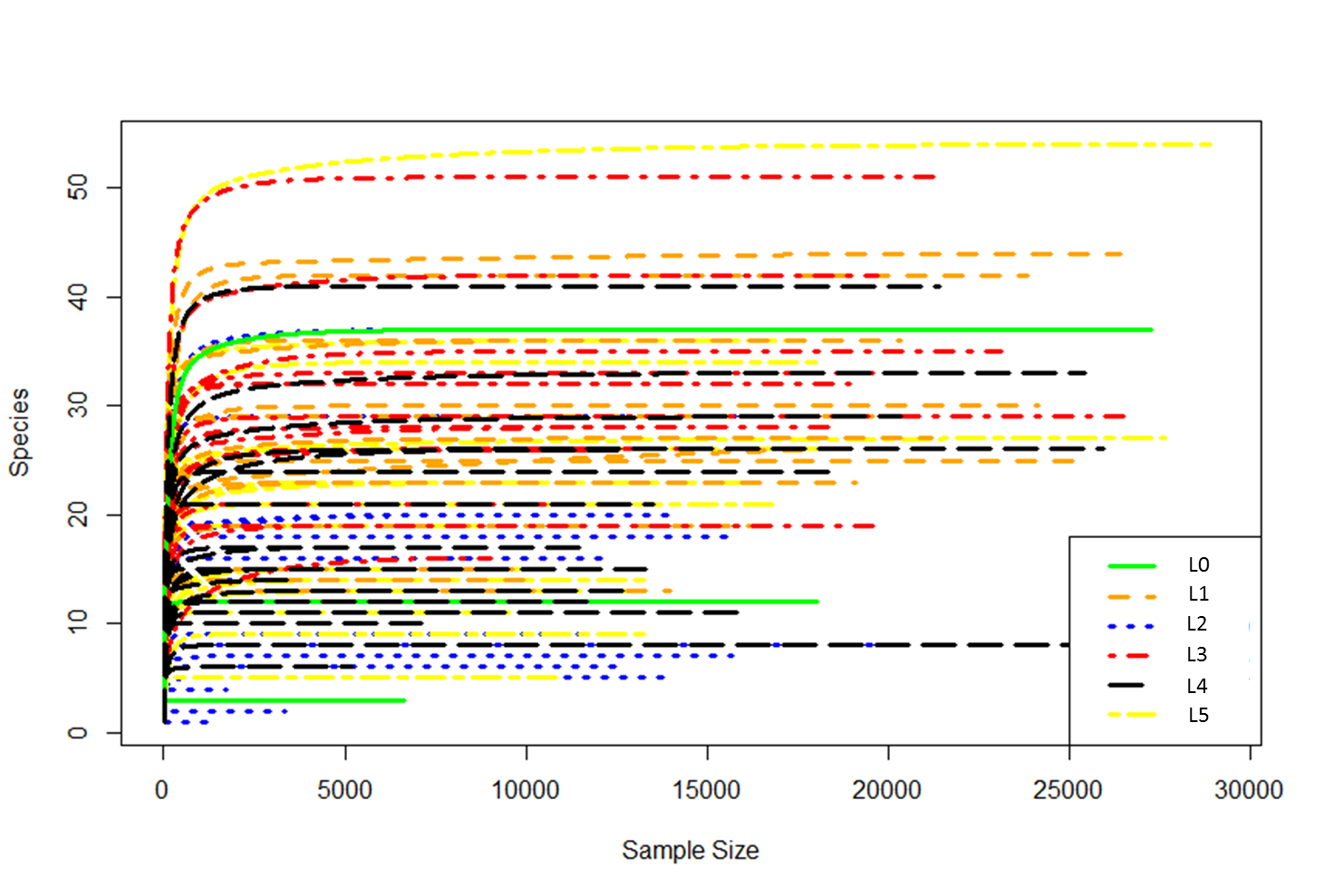

Supplement: Supplementary file 2 — Additional file 2. Rarefaction curves of the sequencing data. The observed species of each sample was in plateaued saturation phase over 1000 reads. Each color indicates a litter and sample size denotes the reads length/per sample. Litters were represented in sequential numbers 0–5. L0 included uninoculated control piglets. Litters 1–5 included all M. hyopneumonaie experimentally inoculated piglets. [file 13567_2019_701_MOESM2_ESM.docx]

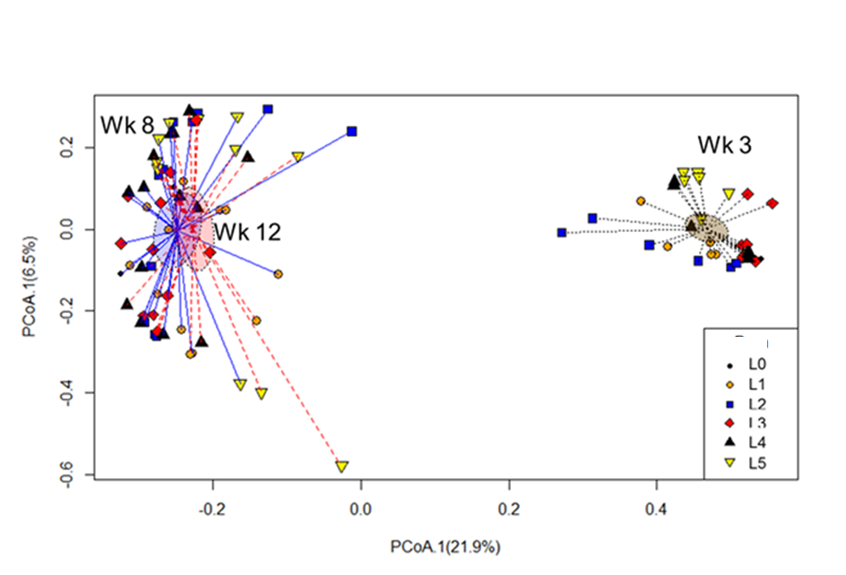

Supplement: Supplementary file 3 — Additional file 3. Difference in bacterial composition among different samples. The PERMANOVA results showed that the microbiome composition in samples was influenced by age (p = 0.01). Litters were represented in sequential numbers 0–5. L0 included uninoculated control piglets. Litters 1–5 included all M. hyopneumonaie experimentally inoculated piglets. [file 13567_2019_701_MOESM3_ESM.docx]

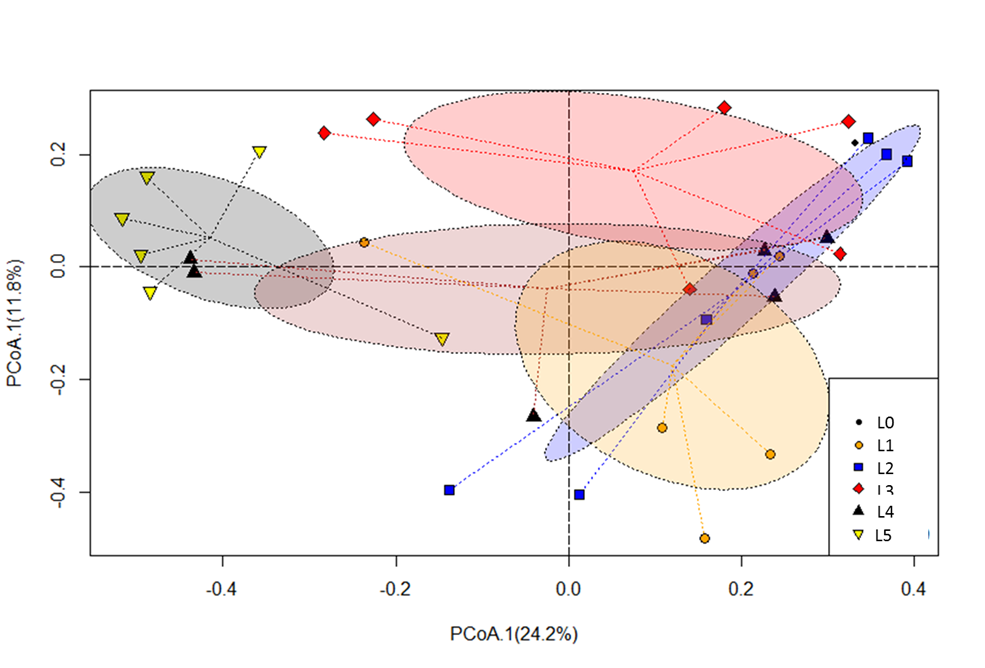

Supplement: Supplementary file 4 — Additional file 4. Samples cluster by litters at week 3 in PCoA plots. Each dashed ellipse represents the 95% confidence interval for the centroid of each stratification group. Litters were represented in sequential numbers 0–5. L0 included uninoculated control piglets. Litters 1–5 included all M. hyopneumonaie experimentally inoculated piglets. Piglets from L3 and L5 showed the least LS whereas those from L2 and L4 showed the highest LS. [file 13567_2019_701_MOESM4_ESM.docx]

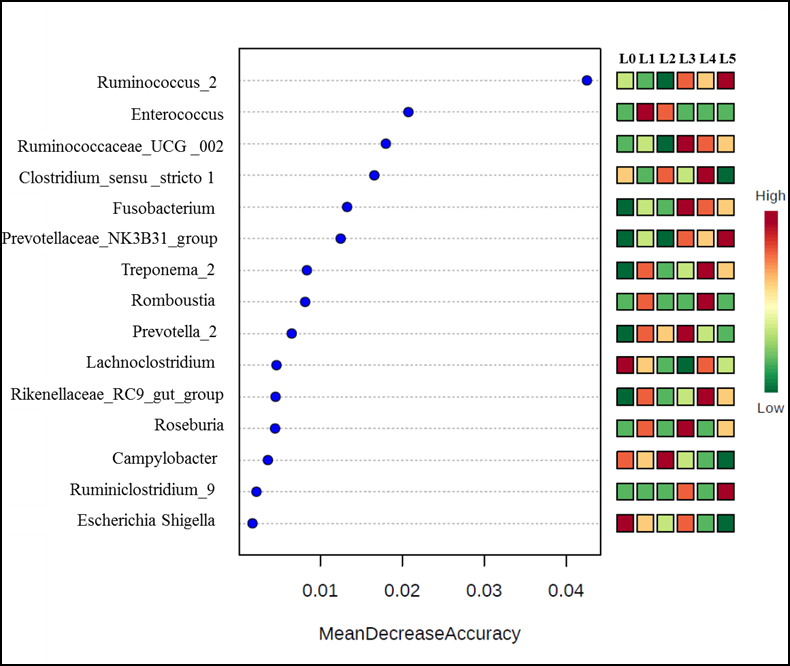

Supplement: Supplementary file 5 — Additional file 5. Differences in phylotypes of early life gut microbiota corresponding to different litters were identified Random Forest analysis. Using the Random Forest classifier, the most discriminative genus-level taxa between litters were identified. The taxa are ranked by the mean decrease in classification accuracy when they are permuted. The mean decrease accuracy is a measure of predictive power. The value indicates how much predictive power is lost if a given genus is removed or permuted in the Random Forest algorithm while classifying samples into litter groups. Litters were represented in sequential numbers 0–5. L0 included uninoculated control piglets. Litters 1–5 included all M. hyopneumonaie experimentally inoculated piglets. [file 13567_2019_701_MOESM5_ESM.docx]
